# Phenotypic traits differentiating the genetic resources of pea (Pisum sativum L.) by the type of use

**DOI:** 10.18699/VJGB-22-74

**Published:** 2022-11

**Authors:** E.V. E.V. Semenova, A.P. Boyko, L.Y. Novikova, M.A. Vishnyakova

**Affiliations:** Federal Research Center the N.I. Vavilov All-Russian Institute of Plant Genetic Resources (VIR), St. Petersburg, Russia; Adler Experiment Station – Branch of the N.I. Vavilov All-Russian Institute of Plant Genetic Resources, Sochi, Russia; Federal Research Center the N.I. Vavilov All-Russian Institute of Plant Genetic Resources (VIR), St. Petersburg, Russia; Federal Research Center the N.I. Vavilov All-Russian Institute of Plant Genetic Resources (VIR), St. Petersburg, Russia

**Keywords:** pea, VIR collection, accessions, trait variability, variability, correlations, ANOVA, PCA, горох, коллекция ВИР, изменчивость признаков, корреляции, дисперсионный анализ, метод главных компонент

## Abstract

The paper presents an analysis of the data obtained for pea accessions from the VIR collection studied at the Adler Experiment Station in the setting of the Krasnodar Territory in 2017–2019. It was for the f irst time that these accessions were studied for a set of phenotypic traits. The object of the study was a sample of 494 pea accessions originated from 43 countries and 18 regions and territories of the Russian Federation. The work was carried out in compliance with the methodological guidelines developed at VIR. Statistica 13.3 software was employed for statistical data processing. An assessment of four qualitative, 10 quantitative and four phenological traits in the accessions made it possible to differentiate them by the type of use, that is, as dry, forage and garden peas. The varieties differing in the type of use signif icantly differed by the values of such traits as stem length, number of pods per plant, number of nodes to the f irst f lower, number of f lowers in the inf lorescence, the maximum number of seeds per pod, pod length, and a narrower pod of forage pea compared to that of dry and garden peas. The average values of these traits were recorded for the peas with different types of use. The maximum difference was noted between garden and forage pea varieties. Dry pea varieties occupied an intermediate position. The complex of phenotypic traits identif ied determines the differences between three types of pea use, which is important when selecting the initial material for breeding appropriate varieties

## Abstract (ru)

Поддержание жизнеспособности и изучение образцов коллекции гороха ВИР осуществляется на опытных станциях института в разных агроклиматических зонах страны. Каждый образец коллекции включен в трехлетний цикл полевого изучения по комплексу селекционно значимых признаков. Фенотипический скрининг коллекции позволяет осуществить оценку признаков, выявить характер их изменчивости и ранжировать образцы по значениям признаков в качестве исходного материала для селекции. Один из основных принципов дифференциации генофонда гороха – разделение образцов по направлениям использования: зерновому, кормовому и овощному. Каждое из этих направлений требует специализированного исходного материала. Между тем четко очерченного перечня различий между фенотипами растений каждого из этих направлений не существует. Более того, имеется множество сходных признаков, порой затрудняющих отнесение сорта к той или иной категории использования. В настоящей статье приведены анализ и обобщение данных трехлетнего фенотипирования образцов коллекции в условиях Краснодарского края на Адлерской опытной станции. Объектом исследования служили 494 коллекционных образца, происходящих из 43 стран и 18 областей и краев Российской Федерации. Образцы оценивали по четырем качественным, десяти количественным и четырем фенологическим признакам. Статистическая обработка данных полевой оценки позволила выявить, что образцы означенных направлений достоверно различались по комплексу признаков: длина стебля; число бобов на растении; число узлов до первого цветка; число цветков в кисти; максимальное число семян в бобе; длина и ширина боба. Определены средние значения этих признаков для всех направлений использования. Овощные сорта максимально отличаются от кормовых. Зерновые занимают промежуточное положение. Выявленный комплекс фенотипических признаков, определяющий различия трех групп направлений использования гороха, упростит работу с коллекцией, в частности подбор исходного материала для селекции соответствующих сортов.

## Introduction

The VIR collection of peas has accumulated the global diver- sity of Pisum sativum L. and contains more than 8 thousand accessions from 93 countries of the world. The collection is structured in accordance with the botanical and agroecological classification, the status of the accessions reflecting the degree of breeding process completeness, and is ranked in accordance with the value of biological and agronomic traits, etc. In order to use the gene pool of peas in the national economy, it is most important to differentiate it according to the types of use as dry, forage, and garden peas.

There is no clearly delineated list of differences between plant phenotypes belonging to each of these three groups. Moreover, there is a multitude of common, so-called “over- lapping” traits, which sometimes make it difficult to attribute a variety to a particular group. Breeding efforts aimed at im- proving varieties of all types of use have common tasks, e. g., high yield, high protein content in seeds, resistance to lodging, plant architectonics suitable for mechanized harvesting, and resistance to pathogens. However, there are complexes of traits characteristic of each group of economic use, some of which are inherent only in one particular group.

Dry peas (P. sativum L. subsp. sativum), which are used as a food crop, are characterized by white flowers, smooth seed surface, mainly with a yellow and yellow-pink seed coat (green and gray-green also possible). For good digestibility, the seed coat must be thin (Khangildin, 1972). Dry pea breed- ing is aimed at high seed productivity, high harvesting index (that is, seed yield in relation to the cut pea mass), and high protein content.

Forage pea varieties have differently colored flowers and seeds of different color, mostly dark and speckled. The breed- ing of mown forage pea varieties is aimed at obtaining high green mass volume, high rate of its accumulation, plant tallness and high leafiness, low percentage of fiber and high protein content in the green mass (Adamova, 1975). There- fore, when breeding forage varieties, it is better to use the forms with the traditional leaf type and indeterminate type of growth (Pea..., 2019). Low 1000 seed weight (less than 100 g) is desirable for forage varieties, as it makes it possible to reduce the weight of the sown seed. Among the varieties of dry and forage peas, there exist transitional forms, which can be called grain-forage varieties.

Garden pea varieties are white-flowered, with brain-like (wrinkled), predominantly green seeds, and large pods. They are required to be uniform in flowering and fruit formation, as well as to have a high yield of green peas in relation to the vegetative mass, which implies a relatively low plant height. One of the main aspects to be pursued in garden pea breed- ing is the improvement of the carbohydrate complex, which determines the taste of green peas in fresh and canned form. This is a high sugar content (6.5–8.5 %) along with a relatively low accumulation of starch (4–5.5 %) containing a high per- centage of amylose (Samarina, 1970; Alikina (Putina) et al., 2016; Putina et al., 2018). Starch grains of garden peas have a specific complex structure with the predominance of small fragments.

A clear differentiation of pea varieties by the type of use is crucial for the characterization of the initial material and its targeted use. This is especially important when diverting from the breeding of universal varieties to breeding varieties for specific use. The identification of criteria for distinguishing leguminous crop varieties by different types of use by phe- notypic traits is carried out in VIR systematically (Vishnya- kova et al., 2011, 2013; Burlyaeva, Malyshev, 2013; Burlyaeva et al., 2014).

The annual regeneration of accessions from the VIR collec- tion and their phenotypic assessment in the field for a number of biological and agronomic traits makes it also possible to reveal the differences within the gene pool according to a variety of parameters along with obtaining the data on the assessed traits. The present paper was aimed at outlining the range of traits that determine the subdivision of the gene pool of pea (P. sativum L.) according to areas of economic use.

## Materials and methods

Material. The collection accessions were studied for three years (2017–2019) at the Adler Experiment Station, a branch of VIR. The study included 494 accessions of peas (P. sati- vum L.) (203 of dry, 217 of garden, and 74 of forage peas) of various types of use introduced in the collection from 43 countries and from 18 regions and territories of the Rus- sian Federation received in the VIR collection since 2005. Ten countries were represented by ten or more accessions; these are Russia (112 accessions), USA (94), France (45), Australia (27), the Netherlands (26), Ukraine (18), China (16), Syria (14), Germany (12), and Canada (10). Within the Russian Federation, most of the accessions came from the Vologda Province (19 accessions), Oryol Province (14), Tyumen Pro- vince (14), Moscow Province (13), Krasnodar Territory (11), and Rostov Province (10).

Methods. The accessions were studied in compliance with the methodological guidelines (Vishnyakova et al., 2010). The seeds were sown in the third ten-day period of March on single-row plots 2.5 m long (~1 m2). A description of phe nological and morphological characteristics was carried out during the vegetation period. The plants were harvested as they matured, from early June through early August. They were gathered in bundles and assessed for their main mor- phological, biological and economic traits. Some parameters were evaluated in points, in accordance with the “Interna- tional Comecon List of Descriptors for the Genus Pisum L.” (Makasheva et al., 1986), while for some the measurement was made in grams, pieces and days.

The list of the assessed traits includes: • Qualitative indicators (points): seed color, leaf morphotype, presence/absence of the parchment layer in the pod valve, fusion of the seed stalk and testa; • Quantitative traits: stem length (cm), number of flowers per inflorescence (pcs), pod length (cm), pod width (cm), number of nodes to the first flower (pcs), yielding ability (seed yield per plot, g), seed productivity per plant (g), 1000 seed weight (g), number of pods per plant (pcs), re- sistance to pea weevil (Bruchus pisorum L.) as percentage of the healthy seeds mass out of the total mass; • Phenologial dates (sowing, emergence, flowering, ripen- ing). Duration has been calculated for the following in- terphase periods (days): from sowing to emergence (SE), from emergence to flowering (EF), from flowering to ripening (FR), and the growing season duration – from emergence to ripening (ER).

The results of quantitative traits assessment are given as average values for three years. Statistical processing employed Statistica 13.3 software. Correlations between quantitative traits of accessions were investigated. The one-way analysis of variance (ANOVA) was carried out for the type of use, presence of a parchment layer, fusion of the seed stalk and testa, and the two-factor ANOVA for the type of use and mor- photype. For post hoc comparisons, Tukey’s test for unequal samples was used. The sample structure was investigated by the principal component analysis (PCA). The study adopted a significance level of 5 %.

Weather conditions during the study. The Experiment Station is located on the northeastern coast of the Black Sea in the Krasnodar Territory with a humid subtropical climate, warm rainy winters and sunny summers. During the period of the study (2017–2019), 2017 was the coldest and wettest year, with the average temperature of 15.9 °С for April–June, and 403 mm of the total precipitation. The year of 2018 was the warmest, with the average temperature of 19.1 °С in April–June, and the total precipitation of 119 mm. The year of 2019 was characterized by an average temperature of 18.5 °C and precipitation of 233 mm for the above-mentioned period. Therefore, in general, the weather conditions at the station are quite consistent with the agroclimatic requirements for pea growing.

## Results and discussion

Phenological data

The average duration of the period from emergence to the onset of flowering ranged from 27 to 53 days for dry peas, from 28 to 54 for garden peas, and from 34 to 58 days for forage pea accessions (Table 1). The earliest onset of flower- ing (on day 27) was recorded for the dry pea variety ‘Nain de Mai’ (k-10068) from France, and on day 28 for the garden pea varieties ‘Salinero’ (k-9811) from the Netherlands and ‘Extra Rapide’ (k-9137) from France. For the bulk of dry pea accessions (>80 %), the onset of flowering was recorded in the interval of 36–45 days from emergence. The distribution of garden pea varieties according to this indicator was more uniform. In forage pea accessions, flowering occurred later.

The most early ripening accession from the studied sample (k-9796, ‘Alsweet’, USA) belonged to the garden pea cate- gory and had the emergence to ripening period duration of 59 days, on an average, while the most late maturing accession (k-10174, ‘Kormovoy-50’, RF, Altai Territory) was a forage pea variety that matured in 84 days (Table 2). A high positive relationship between EF and ER in the entire sample (r = 0.87) should be noted.

**Table 1.**
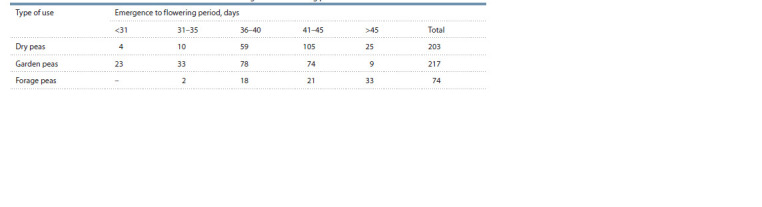
Numbers of accessions with different duration of the emergence to flowering period

**Table 2.**
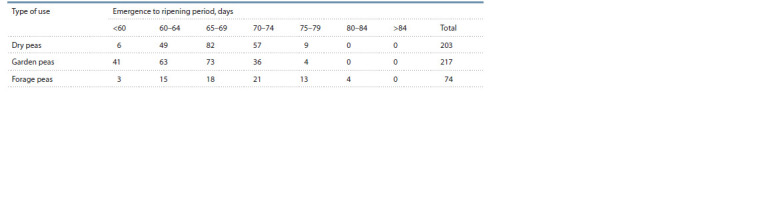
Numbers of accessions with different duration of the emergence to ripening period

Comparison by ANOVA between groups of varieties of different type of use

An analysis of trait values by one-way ANOVA (Table 3) showed that the accessions of different types of use manifested significant differences concerning the majority of the studied traits, except for FR (p = 0.636).

**Table 3.**
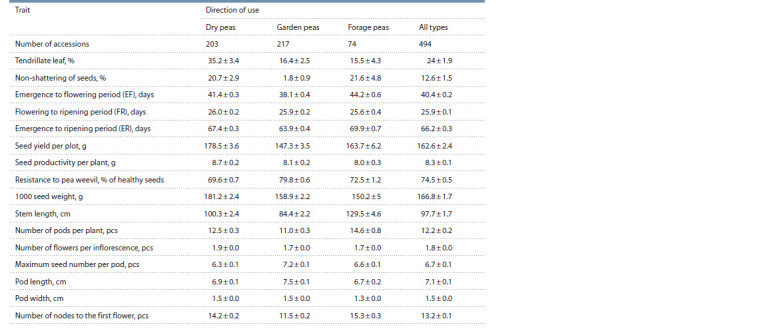
Values of the studied traits in pea groups of different type of use

The leaf morphotype is a significant trait determining suit- ability of a variety for mechanized harvesting. Most modern varieties are semi-leafless (with tendrillate leaf type) (afaf genotype). In the studied sample, this morphotype was sig- nificantly more common in dry peas (35.2 %) than in garden (16.4 %) and forage peas (15.5 %). Such a distribution of accessions is quite consistent with the current state of pea breeding and the requirements to varieties of different types of use. This feature is not relevant for forage varieties, as was mentioned above. Large foliage that ensures abundant vegetative mass can be better achieved with the traditional leaf type. As for garden pea varieties, creation of semi- leafless ones began relatively late both abroad and in this country in comparison with cereals, and is in the process of development (Alikina (Putina) et al., 2016). The absence of significant innovations in the domestic breeding of garden peas is also evidenced by the recently revealed fact that both old and, to even a greater extent, new garden pea varieties are phenotypically less diverse than the foreign ones (Sinjushin, Anisimova, 2020).

The trait of seeds non-shattering due to the fusion of the seed stalk and testa was rarely observed in garden peas (in 1.8 % of varieties) compared with dry (20.7 %) and forage peas (21.6 %). This is explained by the fact that the trait was introduced into pea varieties to prevent seed shedding when ripe pods crack as they dry out (Zelenov, 2013). This is impor- tant for dry pea varieties used for both food and feed purposes. Garden pea varieties are harvested at technical ripeness, long before the possible cracking of the pods, which makes this feature not relevant. In addition, the stalk being firmly adhered to the seed spoils the appearance of canned peas.

The ER period duration averaged 66.2 days for all acces- sions, while all groups were significantly different. The longest duration of the ER period was recorded for forage varieties (69.9 days), medium for dry (67.4 days), and the shortest for the garden pea group (63.9 days). These figures correspond to the purpose of the varieties: the maximum accumulation of the vegetative mass in forage varieties requires a longer period, and garden peas require the minimum period for achiev- ing their technical ripeness. In our opinion, the ER period observed by us for garden peas can be shorter. We explain it by the fact that the sample contained quite many old garden pea varieties. In contrast to them, modern varieties are more early-ripening. For example, modern varieties created at the Krymsk Experiment Breeding Station have a growing season of 53 to 75 days, thereby providing a permanent supply of peas for the long-term and uninterrupted processing (Besedin, 2014; Besedin, Putina, 2019; Putina, Besedin, 2020).

The ER period duration is associated with that of the EF pe- riod, which averaged 40.4 days: it was significantly shorter for garden peas (38.1 days) than for dry (41.4 days) and forage peas (44.2 days), which did not differ significantly between themselves. The FR period for all three groups did not differ and averaged 25.9 days.

The seed yield per plot averaged 162.6 g; dry peas with the highest yield (178.5 g/plot) significantly differed from garden peas (147.3 g/plot). Forage peas had a medium yield value of 163.7 g and did not differ significantly from other groups. The seed productivity per plant did not differ significantly according to Tukey’s test and amounted to 8.3 g.

The pea weevil resistance in the studied sample averaged 74.5 % of healthy seeds. The highest value was demonstrated by garden peas (79.8 %), which was significantly higher than that of dry (69.6 %) and forage peas (72.5 %), which did not differ significantly between themselves. To a certain extent, a lower susceptibility of garden peas is ensured by their early maturity, which makes it possible to avoid the pea weevil flight in the beetle stage. The latter is known to follow a certain seasonal pattern.

The average 1000 seed weight (seed size) in the sample was 166.8 g. Dry peas were found to have the largest seeds (181.2 g), which were significantly bigger than those of gar- den (158.9 g) and forage peas (150.2 g), which did not differ significantly between themselves.

In addition, significant differences between the three groups were noted for the traits listed in Table 3 such as the stem length, the number of pods per plant, the number of nodes to the first flower, the number of flowers per inflorescence, the maximum number of seeds per pod, the pod length and width.

The two-factor analysis of the relationship between the type of use and the morphotype (tendrillate/traditional) em- ployed 116 accessions of the tendrillate and 368 ones of the traditional morphotype. Ten accessions in the sample featured other morphotypes of the leaf: five had acacia-like (tl), one dissected leaflet (af tacA), and four multiple imparipinnate leaf morphotypes (af tl )

**Fig. 1.**
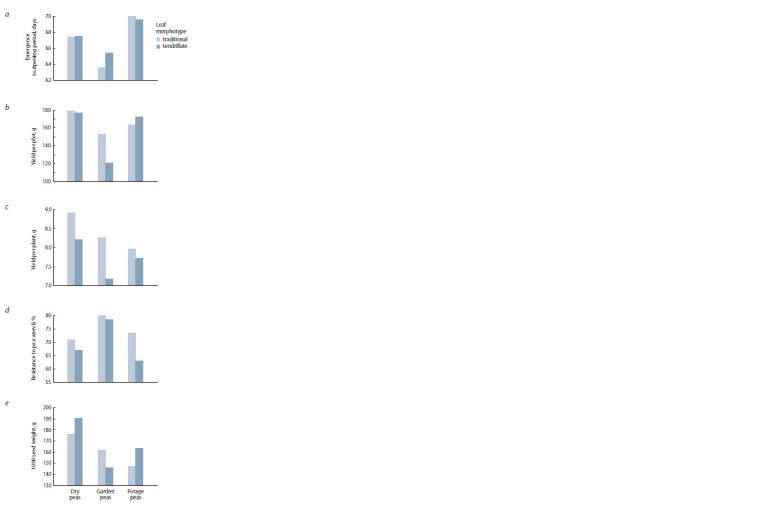
Characteristics of pea accessions of different type of use with tendrillate and common leaf types.

The tendrillate morphotype was characterized by significant differences from the accessions with traditional leaf mor- photype of the leaved forms, regardless of the type of use: by a greater proportion of accessions with non-shattering seeds (25.9 vs. 8.2 %) on an average, which can be explained by the fact that both traits were introduced into varieties in relatively recent times, therefore, the majority of seed shat- tering genotypes is inherent in varieties with the traditional morphotype. The tendrillate leaf varieties are characterized by a lower resistance to pea weevil (70.1 vs. 75.8 %) (Fig. 1), a shorter stem length (84.7 vs. 102.2), fewer pods per plant (10.8 vs. 12.6), and more flowers per inflorescence (2.0 vs. 1.7, except for the forage peas). The seed yield per plot did not differ significantly (159.6 g for the tendrillate pea, and 163.7 g for the traditional morphotypes).

Correlation analysis

The 3-year average values for the varieties were used to cal- culate the correlations of the economically important traits of the accessions with all the studied indicators (Table 4).

**Table 4.**
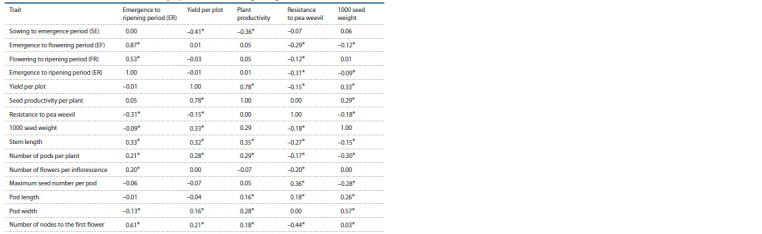
Coefficients of correlation between economically important traits and other agrobiological indicators Coefficients with 0.05 significance level

Medium and strong correlations, i. e. those with the correla- tion coefficient (r) with the module greater than 0.3, have been analyzed.

Economically important characters include such quantita- tive traits as the growing season duration (ER), yield, seed productivity per plant, 1000 seed weight, and resistance to pea weevil. The relationships between traits in the groups of diffe- rent type of use was the same for most characters, which makes it possible to characterize the sample as a whole (see Table 4).

The yield per plot positively correlated with the productivity per plant (r = 0.78), 1000 seed weight (r = 0.33), and the stem length (r = 0.32), while there was a negative correlation with the sowing to emergence period (r = –0.41). The first three relations are obvious, while the last is apparently explained by the fact that the long-emerging seeds have lower germination energy, which is an indicator characterizing simultaneousness and uniformity of seedlings emergence, hence good uniformity and survival of plants, which ensure their productivity.

On an average, plant productivity in the sample was posi- tively associated with plant length (r = 0.35) and negatively with SE (r = –0.36). However, the ways of its formation were different in varieties of different types of use: in dry peas, the coefficient of relationship between seed productivity and the number of pods was r = 0.31. In garden peas, the coefficient of relationship with the stem length was r = 0.53, and 0.49 with the number of pods. In forage varieties, the coefficient of relationship with the stem length was r = 0.40; it was 0.32 with the number of seeds per pod, 0.48 with the pod length, 0.47 with the pod width, and r = 0.48 with 1000 seed weight.

The ER period duration correlated more with that of EF (r = 0.87) than with the FR period duration (r = 0.53).

**Fig. 2.**
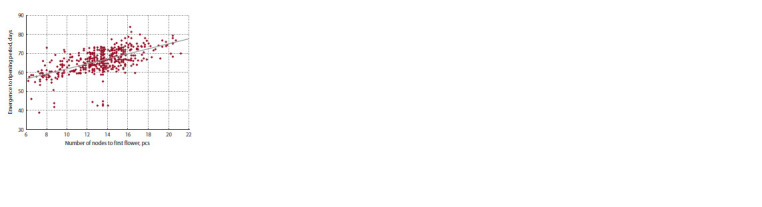
The emergence to ripening period duration as a function of the number of nodes to the first flower.

The relationship between the stem length and the growing season (r = 0.33) is explained by the fact that the bulk of the varieties are indeterminate; the longer a plant lives, the longer it is. A correlation between the ER period and the number of nodes to the first flower (NN) was found to be r = 0.61, which confirms the role of NN as an indicator of early maturity (Makasheva et al., 1986). With an increase in NN by one node, the ER period increases by 1.3 days (Fig. 2). This regularity can be expressed by the formula ER = 48.8 + 1.3 × NN.

**Fig. 3.**
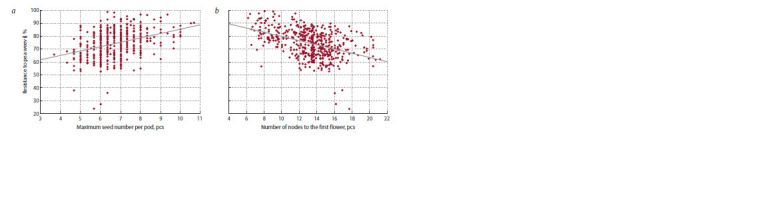
Resistance to pea weevil as a function of seed number per pod (a) and number of nodes to the first flower (b).

In general, pea weevil resistance in the sample was posi- tively associated with the number of seeds per pod (r = 0.36), and according to the type of use, r was 0.32 for dry, 0.22 for garden, and insignificant 0.01 for forage peas. Pea weevil resistance was negatively associated with NN (r = –0.44); according to the type of use, r was –0.29 for dry, –0.40 for garden, and insignificant (–0.05) for forage peas. The bigger the number of unproductive nodes, i. e. the later a variety ripens, the fewer the number of healthy seeds due to a greater damage by the pest. Early ripening accessions avoid the flight of insects; therefore, they get less damaged (Fig. 3). The coef- ficient of relationship with the growing season duration (ER) according to the type of use was r = –0.18 for dry peas, –0.32 for garden, and 0.02 for forage peas.

The percentage of healthy seeds was higher when seeds were green (79.3 %), smaller for yellow seeds (68.8 %), in both dry (73.7 vs. 68.5 %) and garden peas (80.4 vs. 71.9 %). This was also evidenced by the fact of a stronger pea weevil resis- tance in garden pea varieties with predominantly green seeds.

There was a positive relation between 1000 seed weight and the pod width (r = 0.57). This dependence was observed in accessions of all types of use and demonstrated the stron- gest correlation, that is, with r = 0.43 for dry peas, 0.65 for garden, and 0.71 for forage peas. With an increase in the pod width (PW) by 1 cm, 1000 seed weight (W1000) increases by an average of 109 g. This dependence can be expressed by the formula W1000 = 8.7 + 109.0 × PW.

Seed productivity per plant is one of the most important traits for the pea yield structure and, together with 1000 seed weight, it determines the individual productivity of plants. This trait is known as one of the most variable in different crops, including peas.

The highest coefficient of year-to-year variation was ob- served for the seed yield per plot (55.5 % per sample, on an average), while the yield per plant was slightly more stable (36.6 %). The number of pods (28.4 %) and the stem length (14.4 %) demonstrated a greater stability. The number of flowers per inflorescence (2.2 %), NN (4.1 %), pod length (4.4 %), pod width (4.5 %), the maximum number of seeds per pod (7.2 %), and 1000 seed weight (10.3 %) were most stable over the years.

Polymorphism within the sample was subjected to the principal components analysis (PCA). According to the scree criterion, four factors, which explain 69.6 % of the total vari- ance, were distinguished (Table 5).

**Table 5.**
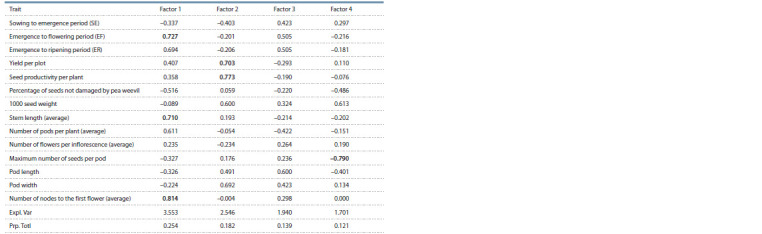
Four factor loadings in PCA The highest loads of the factors are boldfaced

The first factor (explaining 24.5 %) is associated with the ER period duration and such associated characters as the stem length and the number of nodes to the first flower. It can be called the plant vegetation factor. The second factor (18.2 %) is associated with the yield per plot, seed productivity per plant, 1000 seed weight, and the pod width. The third factor (13.9 %) is the pod length, while the fourth (12.1 %) is the maximum number of seeds per pod.

**Fig. 4.**
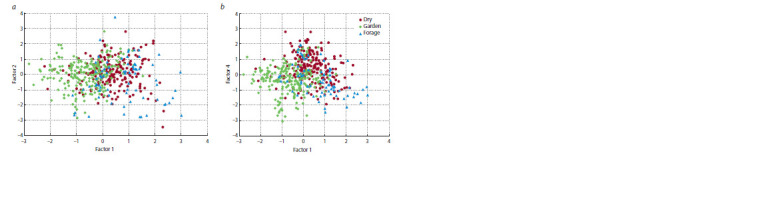
Distribution of 494 pea accessions within the domain of factors 1–2 (a) and 1–4 (b).

The first factor distinguishes the groups of garden and forage type of use (Fig. 4, a), which are opposed in terms of the ER period duration, stem length, and NN. Factors 2 and 3 determine no visual differences between types of use, and according to the fourth factor, the garden type accessions with the maximum number of seeds per pod are contrasted to dry peas with the minimum number of seeds (see Fig. 4, b).

Previously, we studied a sample of 112 pea accessions from the VIR collection in the conditions of the Leningrad Pro- vince and carried out a discriminant analysis of the obtained data, which made it possible to identify the traits by which an accession can be attributed to a particular group of economic use (Semenova, Sobolev, 2009). The traits that were most significant for the statistical attribution of an accession to a variety of use type were such qualitative traits as the seed and pod shape, the presence of anthocyanin in the flower, as well as the range of variability of such quantitative traits as the number of pods, the number of productive nodes per plant, and 1000 seed weight. Like in the present study, the largest seeds were observed in dry pea varieties, smaller ones in garden peas, and the smallest in forage varieties (195.9, 184.3 and 150.5 g, respectively, in the current study, and 181.2, 158.9 and 150.2 g in the previous). Interestingly, the average number of pods per plant was the same in both experiments, i. e. 12.2 pcs, with the largest number for forage peas (14.6 in the present study and 16.5 in the cited work), medium for dry pea varieties (12.5 vs. 11.4), and the smallest for garden peas (11.0 vs. 8.6). Similar results with the current ones were obtained on the basis of the “number of seeds in a bean”, despite the fact that the average number was calculated in the cited work, and we have the average of the maximum number of seeds in a bean. The highest value was noted in vegetable varieties (5.0 in the cited and 7.2 in this work), the average in fodder varieties (4.8 and 6.3) and the minimum in cereals (4.4 and 6.6).

The similarity of the results obtained from the studies carried out in a wide range of ecological and geographical conditions in both experiments indicated that the listed traits can be regarded as differentiating ones when attributing pea accessions to one or another type of economic use.

The RAPD marking of the above-mentioned phenotyped sample of 112 accessions revealed the genetic proximity of varieties within the limits of different types of use, and their distance from each other. The dendrogram of genetic kinship shows the tightly grouped garden pea varieties, and compactly located forage varieties, while both groups were consider- ably remote from each other. Dry pea varieties, which show genetic affinity to both groups, were initial for both of them (Vishnyakova et al., 2011). Like in the present study, it was established that both dry and forage pea varieties contain transitional forms that occupy an intermediate position and can be called grain-fodder varieties.

In the work of French scientists who studied a sample con- taining 148 modern pea varieties of mainly West European origin and primitive forms using 121 protein markers and PCR analysis, the sample was also differentiated by the types of use into dry, forage, and grain fodder peas. It was possible to trace the main tendencies in the West European breeding over the past twenty years of the 20th century, such as an increase in seed size, predominance of white-flowered and semi-leafless forms, and an increase in cold resistance required for sowing in autumn, which is widely practiced in European countries (Baranger et al., 2004).

## Conclusion

A complex of phenotypic traits that significantly differed in pea varieties of different type of economic use (dry, forage and garden) has been revealed. These include the stem length, the number of pods per plant, the number of nodes to the first flower, the number of flowers per inflorescence, the maxi- mum number of seeds per pod, pod length, and a narrower pod of forage peas compared to that of dry and garden peas. The average values of these traits were recorded for peas of all types of use. The largest number of distinctive traits was observed in garden pea varieties, which demonstrated their maximum difference from forage varieties. Dry pea accessions occupy an intermediate position and have a number of traits that overlap with those of forage ones.

A complete description of the material according to the features listed in the article was published in 2020 in the “Catalog of the VIR Global Collection”, issue 910 (Semenova et al., 2020)

## Conflict of interest

The authors declare no conflict of interest.
